# Three-dimensional printed, proximal phalangeal prosthesis with metatarsophalangeal joint arthroplasty for the treatment of a giant cell tumor of the fifth toe: The first case report

**DOI:** 10.1016/j.ijscr.2020.06.069

**Published:** 2020-06-22

**Authors:** Chandhanarat Chandhanayingyong, Korakod Srikong, Chedtha Puncreobutr, Boonrat Lohwongwatana, Rapin Phimolsarnti, Bavornrit Chuckpaiwong

**Affiliations:** a Division of Musculoskeletal Oncology, Department of Orthopaedic Surgery, Faculty of Medicine, Siriraj Hospital, Mahidol University, Bangkok, Thailand; b Biomechanic Research Center, Meticuly Co Ltd., Chulalongkorn University, Bangkok, Thailand; c Advanced Materials Analysis Research Unit, Department of Metallurgical Engineering, Faculty of Engineering, Chulalongkorn University, Bangkok, Thailand; d Biomedical Engineering Research Center, Chulalongkorn University, Bangkok, Thailand; e Division of Foot and Ankle Surgery, Department of Orthopaedic Surgery, Faculty of Medicine, Siriraj Hospital, Mahidol University, Bangkok, Thailand

**Keywords:** Three-dimensional printed prosthesis, Phalangeal tumor, Giant cell tumor of bone, Metatarsophalangeal joint replacement, Case report

## Abstract

**Introduction:**

The majority of patients with bone sarcoma or an aggressive benign tumor of the toe can be successfully treated by amputation. However, limb-salvage surgery for toe tumors remains challenging.

**Presentation of case:**

A 26-year-old female presented with an enlarging mass on her right 5th toe. Imaging studies revealed an expansile osteolytic, destructive lesion of the proximal phalanx of the 5th toe with metatarsophalangeal (MTP) joint invasion. A biopsy specimen confirmed a grade 1, giant cell tumor of the bone. An en bloc resection of the proximal phalanx was performed, and the defect was reconstructed with a patient-matched, three-dimensional, printed titanium proximal phalanx endoprosthesis with an MTP joint extension. The postoperative course was uneventful. The patient has walked with full weight-bearing since early postoperatively. No local recurrence or metastases were evident. However, scar formation occurred after two years, causing an overriding toe deformity.

**Discussion:**

This case represents the first use of a toe prosthesis with MTP joint reconstruction. The complex MTP structure with a preserved metatarsal head facilitates the effort to mimic normal weight-bearing.

**Conclusion:**

A three-dimensional printed prosthesis of the 5th toe is a viable alternative to a bone graft or amputation. However, to avoid stiffness and complications, further study is needed to improve the prosthesis design.

## Highlights

• Customized, single-piece, 3D-printed, titanium phalangeal prosthesis of the 5th toe.
• Replacement of whole proximal phalanx with a mobile joint distally and proximally.
• Patient walks with full weight-bearing, no pain, and no recurrence or metastasis.
• Overriding toe occurred after two years due to scar contracture.
• Prosthesis design development, including size reduction, may improve outcomes.

## 1 Introduction

Giant cell tumors of the bone (GCTB) in the small bones of the hands or feet are rare, accounting for 1.7%–5.4% of all GCTBs [[1], [2], [3], [4], [5], [6]]. They exhibit unique clinical features, including a predominance in females and younger patients and a more aggressive behavior than GCTBs of the large bones [2,4]. This condition can be successfully treated with extended curettage and cementation, bone grafting, or synthetic bone placement [7,8]. However, for GCTB with massive cortical destruction and soft tissue extension (Campanacci type 3), wide resection has been the standard choice of treatment [8]. Human monoclonal antibody against RANKL (denosumab) is indicated in patients with unresectable GCTB. However, denosumab may associate with a doubtful treatment end time [9,10] and high costs, especially in developing countries.

A limited number of limb-salvage procedures have been reported for GCTB of the phalangeal bone of the hand and foot [1,2,4,6,8]. Biological reconstructions following phalangeal resection of a GCTB commonly use an iliac crest autograft or allograft with fusion [8,11]. These procedures may associate with complications requiring long-term immobilization, non-weight-bearing, and the potential for graft resorption and instrument failure. Arthroplasty of the small bones of the hands and feet has limited availability. Most prostheses are designed for arthritic patients and do not provide a solution for segmental or osteo-articular bone loss from a tumor resection.

Recently, three-dimensional (3D) printing technology, capable of precisely reconstructing bone defects, has been successfully used in the orthopaedic field [[12], [13], [14], [15]]. To our knowledge, there are no reports on 3D-printed prostheses of the toe proximal phalanx with single-piece connection of metatarsophalangeal arthroplasty.

We report the surgery and outcomes of a total proximal phalangeal resection of the 5th toe, which was reconstructed with a 3D-printed titanium phalangeal prosthesis with finite element study.

## 2 Presentation of case

**Fig. 1:**
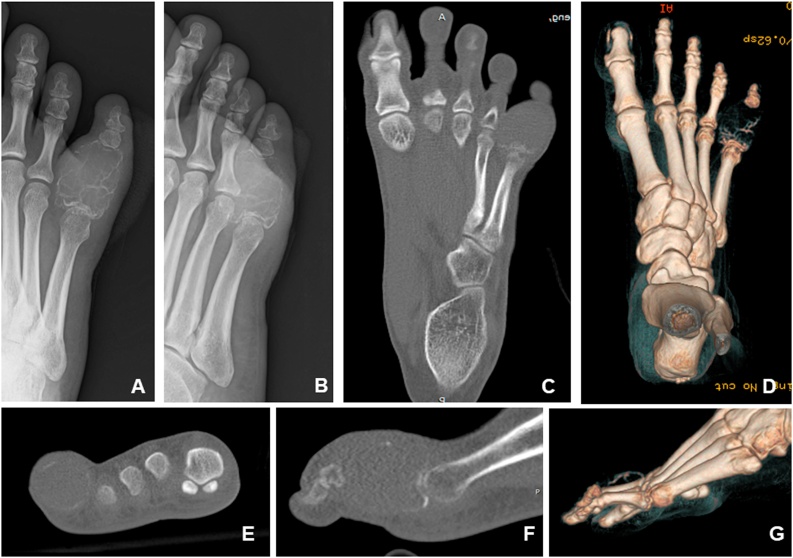
Preoperative imaging. Radiographs (Fig. 1A and B) show an expansile, osteolytic lesion of the proximal phalanx of the right fifth toe. Computed tomography (CT and 3D CT; Fig. 1C–G) show destruction of the cortex with soft tissue expansion and intra-articular invasion.

A previously healthy, 26-year-old female presented with a 2-year history of right 5th toe pain and progressive swelling, but walked well with normal gait. Radiographs revealed expansile geographic osteolytic lesions of the proximal phalanx of the right 5th toe (Fig. 1). Open biopsy confirmed a diagnosis of GCTB. No distant metastasis was detected. She did not take medication regularly and denied family history of genetic disorders. Patient understood her health condition and agreed to undergo wide resection and reconstruction with customized, 3D-printed toe prosthesis after critical discussion of possible methods of treatment. Denosumab was not administered preoperatively.

## 3 Prosthetic design and manufacture

**Fig. 2:**
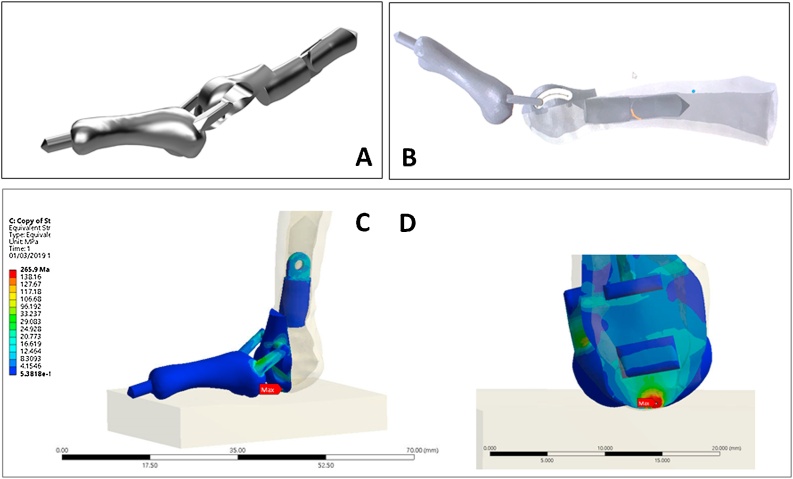
Graphics representing the 3D-printed, personalized, proximal phalanx prosthesis. The posteromedial (2A) and lateral (2B) views of the implant are illustrated. A finite element study showed the maximum yield stress of prosthesis was 900 MPa, from which the calculated Von Mises stress for both 160 N and 800 N at 90° MTP joint extension were 265.9 and 1221.7 MPa. For 160 N loading to toe prosthesis at 90° MTP extension, it caused stress less than the yield stress. For 800 N loading, which reflected the total weight-bearing, it caused stress 1.5 times the maximal yield stress.

The prosthesis, custom-made by an Electron Beam Melting 3D printer (Concept Laser Mlab, Lichtenfels, Germany), was based on the contralateral side of the patient’s 5th toe on a CT scan. It was designed to replace the whole proximal phalanx, with connection to the 5th metatarsophalangeal (MTP) joint to reduce cartilage wearing (Figs. 2A, 2B). Only one-third of the superior part of the metatarsal head was removed. This allowed insertion of the metatarsal stem while facilitating normal weight-bearing via the remaining two-thirds of the metatarsal head.

For the finite element study (Fig. 2C, D), the 3D model was established by Mimics 18.0 and processed in a stereolithography format. Ansys Mechanical 19.2 software was used with Von Misses criteria. The stress distribution on 3D prosthesis and the strain to the 5th MTP joint were analyzed by loading forces to the MTP extension at 90 degrees by 160 N (force on normal walking) and 800 N (total body weight). The results ensured that our endoprosthesis could tolerate the axial loading and shearing force.

## 4 Surgical procedure

**Fig. 3:**
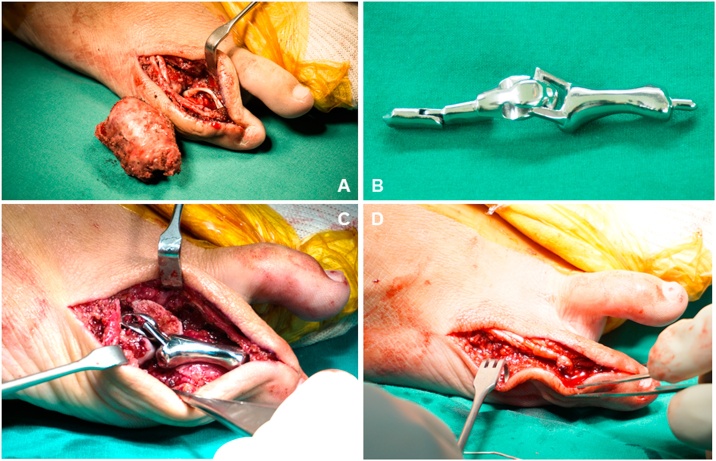
Intraoperative procedures. Wide resection of the GCTB of the proximal phalanx of the 5th toe, with preservation of the lateral hood of the extensor tendon (Fig. 3A). Customized 3D toe prosthesis, sterile and ready to use (Fig. 3B). Surgical application of the prosthesis to the metatarsal bone of the 5th toe with metatarsal stem (press fit; cementless; Fig. 3C). Final reattachment of the extensor tendon and soft tissue coverage (Fig. 3D).

**Fig. 4:**
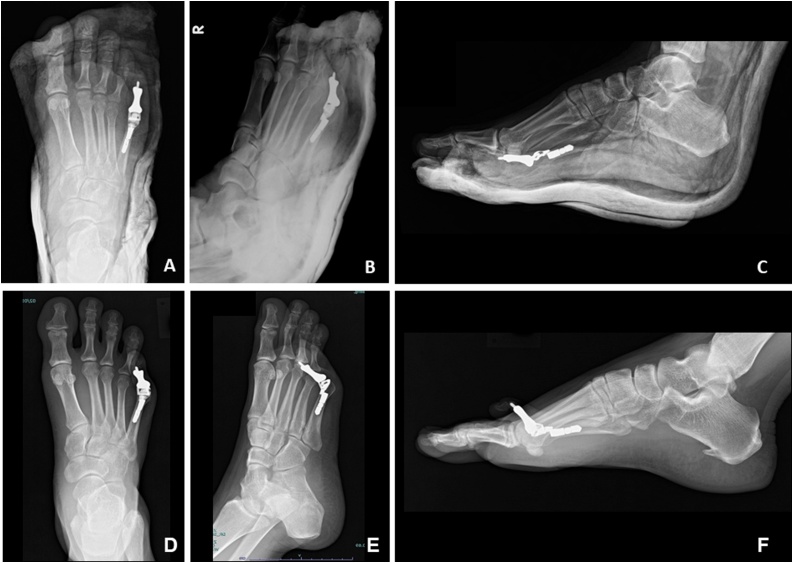
Immediate postoperative images. Anteroposterior (4A), oblique (4B), and lateral (4C) radiographs of the right foot show proper prosthesis placement. Subsequently, overriding toe occurred in two years and evidenced in AP (4D), oblique (4E) and lateral (4 F) radiographs due to scar contracture.

Surgery was performed by orthopaedic oncologist (CC), using the dorsal approach. The dorsal slip and lateral hood of the extensor tendon was retracted medially, enabling access to the entire proximal phalanx (Fig. 3). The neurovascular bundle was protected and retracted inferiorly. Removal of the whole proximal phalanx of the 5th digit with GCTB was done with no local adjuvant therapy. The superior one-third of the metatarsal head of the 5th toe was cut, and the cancellous bone was removed by high speed burr to allow implant insertion. The prosthesis was pressed fit without polymethyl methacrylate. An intraoperative X-Ray was done to ensure proper positioning. The extensor tendon was re-sutured to the distal part. Intraoperative and postoperative radiographs showed the near anatomical position of the MTP joint and proximal phalanx (Fig. 4).

## 5 Follow-up

**Fig. 5:**
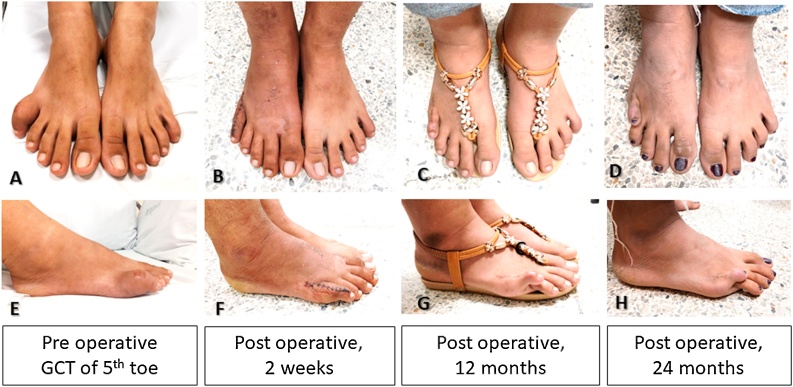
Preoperative pictures of the GCTB of the right 5th toe demonstrated an enlarging mass (5A, 5E). Two weeks after resection and reconstruction with the 3D toe prosthesis, the 5th toe appeared normal in size, length, and alignment (5B, 5F). Dorsiflexion contracture was evident at one year postoperatively (5C, 5G). After 2 years, a fibrotic scar formed and retracted the MTP joint to hyperextend to 40° with an overriding toe (5D, 5H).

A short-leg posterior slab with a toe plate was applied for two weeks after surgery. Weight-bearing was then permitted gradually to full weight-bearing over three months. During the first 4–5 months, the postoperative course was uneventful (Figs. 5A, B). The patient returned to work and wore boots to stand for 8 h a day. The patient walked well on bare feet without support. No pain medication was taken. However, 6 months postoperatively, scar contraction was evident. The MTP joint had both sagittal and transversal stability, but the active motion range of the 5th metatarsophalangeal joint was restricted to 5° plantar flexion and 25° dorsiflexion (35% and 15% losses, respectively, compared to the normal side; Fig. 5C). Dorsiflexion malalignment due to extension contracture occurred, and crossover toe deformity gradually appeared a year postoperatively (Figs. 4D–F, 5). Although this was managed with massage, the method did not seem to soften the scar or reduce the degree of hyperextension. The American Orthopaedic Foot and Ankle Society [16] MTP-interphalangeal for the lessor toes scale score for this patient was 85 (pain, 40; function, 31; alignment, 15). The Musculoskeletal Tumor Society scoring was 25 (83%: no pain, 5; minimal restriction of function, 4; emotional acceptance, 3; no-gait support, 5; intermediate range of walking, 4; normal gait, 4). Serial radiographs showed an intact prosthesis without loosening. The patient reported no pain or difficulty with daily living. Patient declined further surgery, such as soft tissue correction or toe amputation. She also confirmed that if she could choose again, she would still have the 5th toe reconstructed with the metal prosthesis rather than amputation, despite the subsequent dorsiflexion deformity.

This study has been reported accordingly with the SCARE 2018 criteria [17] and submitted to www.researchregistry.com with the unique identifying number (UNI) of researchregistry5556 (https://www.researchregistry.com/browse-the-registry#home/registrationdetails/5eaae5e79cc0590016bbff7d/).

## 6 Discussion

GCTB of the metatarsal or toe phalangeal bone often presents as an advanced stage and requires extensive en bloc resection. [4,6,8] Reconstruction of this area is challenging and often resulting in digit or ray amputation [2,11]. Recently, 3D motion analysis of the foot shows flexion of the 5th MTP joint facilitates normal gait during “push-off”. [18,19] In the present study, we described a novel technique of reconstruction utilizing a patient-matched, 3D printed, titanium proximal phalangeal prosthesis.

The use of such a prosthesis offers the following advantages:

(1) The ability to manufacture a custom-made endoprosthesis with a mobile joint attached accurately from CT data (not previously possible).
(2) The prosthesis is connected in two places: distally to the middle and distal phalanx with a smooth knob, and proximally, with MTP joint arthroplasty, using a key-ring design (Fig. 2B). This provides maximal MTP flexion while the patient walks, and preserves the lower part of the metatarsal head to ensure weight-bearing with the patient’s own bone.
(3) The proximal stem is divided in two to allow passage through the small entry point of the MT head and easy gliding into the metatarsal canal.
(4) Titanium alloy is used due to its weight, strength, and biocompatibility.
(5) The ready-made, anatomically matched prosthesis reduces the intraoperative time.

The prosthetic design development and manufacture took 4 weeks, including 3 minor revisions, a finite element study, and cadaveric surgical fitting.

The patient achieved satisfactory clinical outcomes with normal weight-bearing of the 5th metatarsal head. There were no symptoms of irritation. However, the patient showed dorsiflexion contracture of the 5th toe after 8 months and gradually developed a full-fixed, overriding toe deformity at 18–24 months. To prevent excessive contracture, the authors suggest reducing the size of the 3D printed prosthesis by 10–15% or tightening the loop of the MTP joint to preclude hyperextension deformity. Neutral-position casting is recommended, but beware not to push the phalanx proximally. Early recognition and prompt treatment (massage or serial splinting) should be initiated should this occur.

Longer follow-up is needed to ascertain if soft-tissue stiffness affects the foot function. However, we can report that this method is an effective alternative for toe reconstruction because of its less invasive nature without donor site morbidity. Further study of 3D-printed prosthetic designs, especially in the rare area, is needed to facilitate limb-sparing surgery and reduce complications.

## 7 Conclusions

We reported the novel 3D-printed, proximal phalanx toe prosthesis with total arthroplasty of the 5th MTP joint reconstruction. This is significant because it is the first model of a customized endoprosthesis with a connecting mobile arthroplasty that can be reproduced by a simple 3D printer, among other 3D prostheses which only replace the bone without joint reconstruction. The short-term clinical outcomes were satisfactory, with early rehabilitation. However, scar contracture led to an overriding toe 2 years postoperatively. Although 3D-printed prosthesis for the toe can be considered as an option for the treatment of toe tumors, longer follow-ups and methods to reduce scar formation should be studied to improve treatment quality.

## Conflicts of Competing Interest

No conflicts of interest to declare.

## Source of funding

Decision to submit the manuscript for publication was supported by Siriraj Research Fund, Faculty of Medicine, Siriraj Hospital, Mahidol University.

## Ethics approval

This study was exempt from our institutional review board process due to its retrospective design and as the research involved benign behavioural intervention

## Consent

Written, informed consent was obtained from the patient for this article and the online publication of this case report and accompanying images.

## Author contribution

**Chandhanarat Chandhanayingyong**: Conceptualization; Data curation; Formal analysis, Writing - original draft; Writing - review & editing, Project administration; Researchregistry funding

**Korakod Srikong**: Investigation, prosthesis 3D design, manufacture, finite element study

**Chedtha Puncreobutr**: Supervision, prosthesis 3D design, manufacture, finite element study

**Boonrat Lohwongwatana**: Supervision, prosthesis 3D design, manufacture, finite element study

**Rapin Phimolsarnti**: Supervision

**Bavornrit Chuckpaiwong**: Foot and ankle mechanical consultation, Writing - review & editing

## Registration of research studies

Research registry UIN: researchregistry5556.

## Guarantor

Chandhanarat Chandhanayingyong.

## Provenance and peer review

Not commissioned, externally peer-reviewed.
